# Formulation of a mmaA4 Gene Deletion Mutant of *Mycobacterium bovis* BCG in Cationic Liposomes Significantly Enhances Protection against Tuberculosis

**DOI:** 10.1371/journal.pone.0032959

**Published:** 2012-03-19

**Authors:** Steven C. Derrick, Dee Dao, Amy Yang, Kris Kolibab, William R. Jacobs, Sheldon L. Morris

**Affiliations:** 1 Center for Biologics Evaluation and Research, United States Food and Drug Administration, Bethesda, Maryland, United States of America; 2 Howard Hughes Medical Institute, Albert Einstein College of Medicine, Bronx, New York, United States of America; University of Texas at Tyler, United States of America

## Abstract

A new vaccination strategy is urgently needed for improved control of the global tuberculosis (TB) epidemic. Using a mouse aerosol *Mycobacterium tuberculosis* challenge model, we investigated the protective efficacy of a *mmaA4* gene deletion mutant of *Mycobacterium bovis* BCG (*ΔmmaA4*BCG) formulated in dimethyl dioctadecyl ammonium bromide (DDA) – D(+) trehalose 6,6 dibenenate (TDB) (DDA/TDB) adjuvant. In previous studies, deletion of the *mmaA4* gene was shown to reduce the suppression of IL-12 production often seen after mycobacterial infections. While the non-adjuvanted *ΔmmaA4*BCG strain did not protect mice substantially better than conventional BCG against a tuberculous challenge in four protection experiments, the protective responses induced by the *ΔmmaA4*BCG vaccine formulated in DDA/TDB adjuvant was consistently increased relative to nonadjuvanted BCG controls. Furthermore, the *ΔmmaA4*BCG-DDA/TDB vaccine induced significantly higher frequencies of multifunctional (MFT) CD4 T cells expressing both IFNγ and TNFα (double positive) or IFNγ, TNFα and IL-2 (triple positive) than CD4 T cells derived from mice vaccinated with BCG. These MFT cells were characterized by having higher IFNγ and TNFα median fluorescence intensity (MFI) values than monofunctional CD4 T cells. Interestingly, both BCG/adjuvant and *ΔmmaA4*BCG/adjuvant formulations induced significantly higher frequencies of CD4 T cells expressing TNFα and IL-2 than nonadjuvanted BCG or *ΔmmaA4*BCG vaccines indicating that BCG/adjuvant mixtures may be more effective at inducing central memory T cells. Importantly, when either conventional BCG or the mutant were formulated in adjuvant and administered to SCID mice or immunocompromised mice depleted of IFNγ, significantly lower vaccine-derived mycobacterial CFU were detected relative to immunodeficient mice injected with non-adjuvanted BCG. Overall, these data suggest that immunization with the *ΔmmaA4*BCG/adjuvant formulation may be an effective, safe, and relatively inexpensive alternative to vaccination with conventional BCG.

## Introduction

Despite being an ancient disease, TB remains an enormous public health concern in the 21^st^ century. One-third of the world's population is thought to be infected with *Mycobacterium tuberculosis* and new infections likely occur every second. In 2009, the World Health Organization estimated that there were 14 million active cases of TB, 9.4 million new cases, and 1.7 million deaths due to this disease [1]. A driving force for the resurgent TB epidemic has been the HIV/AIDS pandemic. It has been estimated that approximately 1.5 million individuals are co-infected with TB and HIV and more than 400,000 co-infected persons die each year [2]. Importantly, control of this epidemic has been further confounded by the emergence of multiple drug resistant and extensively drug-resistant *M. tuberculosis* strains which often limit treatment options and make appropriate medical interventions challenging [3].

The only licensed vaccine against TB, *M. bovis* BCG, has been given to over 3 billion persons during its eight decades of clinical use. Randomized controlled trials and retrospective case control studies have shown that BCG immunization is effective in reducing cases of severe disseminated tuberculosis (TB meningitis and miliary TB) in children [4], [5]. Surprisingly, recent studies have also suggested that BCG vaccination may also reduce the risk of childhood infection by *M. tuberculosis* [6]–[8]. However, the effectiveness of BCG vaccine in preventing the most contagious and prevalent form of disease, pulmonary TB, is unclear. BCG-induced protection against TB has been highly variable with protective efficacies ranging from 0–80% in numerous clinical trials [4], [9]. Furthermore, the protection induced by BCG vaccination is often not highly persistent and a substantial waning of the protective responses is generally seen during the first decade after immunization [10]. Given the devastation of the global TB epidemic, the sub-optimal effectiveness of BCG immunization has created a public health urgency to generate an improved TB vaccination strategy.

Since BCG is widely used in areas where the TB burden is high and BCG immunization does reduce the incidence of severe extrapulmonary disease in children, the current focus of many TB vaccinologists has been the development of an approach to amplify BCG-induced immune responses by boosting with viral vectored or subunit TB vaccines after the priming BCG immunization [11], [12]. Although the prime/boost strategy is clearly promising, the complexities of prime/boost immunization schedules and the cost of subunit vaccines may limit its utility in developing countries. Alternative simpler and less expensive approaches may be required in the development of an improved TB vaccination strategy for the developing world. At least two of these TB vaccine improvement approaches have concentrated on modifying conventional BCG vaccine formulations. For example, encapsulation of BCG in lipid preparations has been shown in mice, badgers, guinea pigs, and cattle to improve the immunogenicity and protective efficacy of BCG immunization [13]–[18]. Lipid encapsulation of BCG seems to increase the extent and kinetics of BCG infection at specific immune sites including the lymph nodes. Secondly, BCG strains are being modified to enhance their immunogenicity [19]–[20]. Interestingly, more immunogenic mycobacterial strains have been generated using genetic approaches to remove mycobacterial genes known to inhibit host pro-inflammatory responses. Recently, Dao and colleagues have shown that a deletion in the *mmaA4* gene (which encodes a methyl transferase involved in mycolic acid synthesis) of *M. tuberculosis* removed the selective repression of IL-12 synthesis caused by mycobacterial infections. As a result, the *ΔmmaA4* mutant strain induced significantly elevated levels of this critical Th1-type cytokine in macrophage cultures [21].

Here we have combined two BCG modification approaches in the evaluation of a novel immunization strategy against TB. Specifically, we created a *ΔmmaA4* mutant of BCG and formulated it in the DDA/TDB cationic liposomal adjuvant. This adjuvant has been shown to be safe and immune enhancing in human clinical trials, [22]. We then evaluated the immunogenicity, safety, and effectiveness of this preparation in a mouse model of pulmonary TB. Our data suggests that this mutant BCG/adjuvant formulation induces higher levels of mycobacterial-specific multifunctional T cells, is more protective than BCG vaccine, and, surprisingly, may be safer than BCG when used in immunocompromised animals.

## Materials and Methods

### Animals

C57BL/6 female mice and SCID mice (B6.CB17-Prkdc<scid>SzJ; # 25938) that were 6–8 weeks of age were obtained from the Jackson Laboratories (Bar Harbour, Maine). All mice used in this study were maintained under appropriate conditions at the Center for Biologics Evaluation and Research, Bethesda, MD. This study was done in accordance with the guidelines for the care and use of laboratory animals specified by the National Institutes of Health. This protocol was approved by the Institutional Animal Care and Use Committee of the Center for Biologics Evaluation and Research under Animal Study Protocol 1993-09.

### Preparation of vaccines

The *ΔmmaA4*BCG mutant was derived from BCG Pasteur as previously described [21]. Wild-type BCG Pasteur or the *ΔmmaA4*BCG strain were administered subcutaneously (s.c.) in PBS or adjuvant at 1×10^6^ CFU per immunization in 0.2 ml. The adjuvant- containing vaccines were prepared by mixing the BCG or the mutant BCG with dimethyl dioctadecyl-ammonium bromide (DDA, Kodak, Rochester, NY) and D-(+)-Trehalose 6,6′-Dibehenate (TDB, Avanti Polar Lipids, Alabster, AL). The DDA solution was prepared by heating 25 mg in 10 ml water at 80°C for 20 min and vortexing every 5 minutes. The TDB solution was prepared by adding 1.0 ml of water with 2 µl DMSO (0.2% final) to a vial containing 5.0 mg TDB. The TDB suspension was sonicated until it became homogenous. The adjuvanted vaccines were prepared by mixing 5×10^6^ CFU of BCG or the mutant BCG strain with 0.6 ml of DDA with sufficient PBS to bring the volume to 0.9 ml. One-tenth ml of TDB was added to the BCG-DDA mixture, vortexed three times and then incubated at room temperature for 1 hour. Mice received either one immunization or three immunizations 2 weeks apart.

### Evaluation of vaccine-induced protective immunity in a murine model of pulmonary TB

At 2 months after the final immunization, five mice per group were infected with *M. tuberculosis* Erdman by aerosol at a concentration known to deliver about 200 CFU in the lungs over a 30-minute exposure in a Middlebrook chamber (Glas Col, Terre Haute, IN) [23]. At each time point, the lungs and spleens were homogenized separately in PBS with 0.05% Tween 80 using a Seward Stomacher 80 blender (Tekmar, Cincinnati, OH). The homogenates were serially diluted in PBS +0.05% Tween 80 and plated on Middlebrook 7H11 agar (Difco) plates containing 10% OADC enrichment (Becton Dickinson, Sparks, MD) medium, 10 µg/ml ampicillin, 50 µg/ml cycloheximide, and 2 µg/ml 2-thiophenecarboxylic acid hydride (TCH) (Sigma). The addition of TCH to the agar plates inhibits BCG growth but has no effect on *M. tuberculosis* growth. Plates were incubated at 37°C for 17 days before counting to determine the number of mycobacterial colony forming units (CFU) per organ.

### Evaluation of the safety of BCG or *ΔmmaA4*BCG with or without adjuvant in immunocompromised mice

To evaluate the safety of the different BCG vaccine preparations, we used either mice treated with anti-IFNγ neutralizing antibody or SCID mice. For the studies in mice receiving the anti-IFNγ antibody treatment, mice received intraperitoneal injections of anti-IFNγ (XMG.6) (0.5 mg) at 4 and 2 days prior to receiving one s.c. injection of 1×10^6^ CFU of BCG or *ΔmmaA4*BCG with or without adjuvant. The mice received antibody treatments every 10 days and were then sacrified one month after being infected for evaluation of spleen bacterial burdens. SCID mice received one 10^6^ CFU dose of the different BCG vaccine preparations intravenously (i.v.) and were then sacrificed one month later to quantify bacterial CFU in the spleens.

### Flow Cytometry

Four to five unchallenged mice were used to determine the frequency of CD4+ or CD8+ multifunctional T cells (MFT cells) induced for each vaccine at 2 months post-immunization. Unvaccinated (naïve) mice served as the negative control. Spleen cells from naïve and vaccinated mice were isolated by disrupting the spleens using a 3cc syringe barrel in complete DMEM (cDMEM) consisting of 10 mM HEPES, 2.0 mM L-glutamine, 0.1 mM MEM non-essential amino acids with 10% fetal bovine serum (FBS). After passing the spleen homogenates through a 70 µm cell strainer, the resulting single cell suspension was washed with cDMEM and treated for 1 min with 5.0 ml ACK lysing buffer (Lonza, Walkersville, MD). After washing the spleen cells with an equal volume of media, the cells were resuspended in cDMEM and added to wells of a 24-well plate at a density of 2.5×10^6^ cells per well in 1.0 ml. For measurement of antigen-specific responses, BCG Pasteur was added to the wells at a multiplicity of infection (MOI) of 0.5 bacilli per spleen cell. Wells which contained only spleen cells served as unstimulated controls. After an overnight incubation, Golgiplug (BD Biosciences, San Jose, CA) was added (1 µl per well) to the spleen cells and incubated 4 hours. Unbound cells were removed from the wells and transferred to 12×75 mm tubes, washed with PBS and resuspended in 50 µl PBS. Live-Dead stain (Invitrogen, Carlsbad, CA) (10 µl of a 1∶100 dilution) was added to each tube and incubated for 30 min. at 4°C to allow for gating on viable cells. After washing the cells with PBS-FBS, antibody against CD16/CD32 (FcγIII/II receptor, clone 2.4G2) (Fc block) was added in a volume of 50 µl and incubated at 4°C for 15 min. The cells were then stained for 30 min. at 4°C by adding antibodies against the CD4 (rat anti-mouse CD4 Alexa Fluor 700 [AF-700] Ab, clone RM4-5), and CD8 (rat anti-mouse CD8 peridinin chlorophyll protein complex [PerCP] Ab, clone 53-6.7) proteins at 0.1 and 0.4 µg per tube respectively. Following the incubation, the cells were washed twice with PBS and then fixed for 30 min. at 4°C with 2% paraformaldehyde in PBS. After fixing, the cells were pelleted, washed twice with PBS-FBS and stored at 4°C. Fixed cells were washed twice with perm-wash buffer (1% FBS, 0.01 M HEPES, 0.1% saponin in PBS) followed by intracellular staining using the following antibodies at 0.2 µg per tube: rat anti-mouse IFNγ allophycocyanin [APC] Ab, clone XMG1.2; rat anti-mouse TNFα fluorescein isothiocyanate [FITC] Ab, clone MP6-XT22; rat anti-mouse IL-2 phycoerythrin [PE] Ab, clone JES6-5H4. The cells were incubated at 4°C for 30 min., washed twice with perm-wash buffer and then twice with PBS-FBS. All antibodies were obtained from BD Biosciences.

The cells were analyzed using a LSRII flow cytometer (Becton Dickinson) and FlowJo software (Tree Star Inc., Ashland, Oregon). We acquired 250,000 events per sample and then, using FlowJo, gated on live, single cell lymphocytes. To determine the frequency of different populations of MFT cells, we gated on CD4 or CD8 T cells staining positive for TNFα and IFNγ, TNFα and IL-2, IFNγ and IL-2 or all three cytokines.

### Median fluorescence intensity (MFI) assessments

The MFI for IFNγ or TNFα for CD4 and CD8 monofunctional and MFT cells was evaluated in the different vaccine groups using the FlowJo software. For this study, the MFI is the fluorescence intensity value representing the middle number of the distribution of CD4 and CD8 T cells secreting only IFNγ or TNFα, secreting both IFNγ and TNFα or cells secreting IFNγ, TNFα and IL-2. The data are presented as the mean of the individual MFI assessments for 4–5 mice. The integrated MFI metric (iMFI) was determined by multiplying the mean MFI values for a specific T cell subset by its frequency.

### Statistical analysis

The Graph Pad Prism 5 program was used to analyze the data for these experiments (Graph Pad Software, San Diego CA). The protection data, the CD4 and CD8 T cell flow cytometry results, and the MFI data were evaluated using t test analysis. The correlations between the iMFI values for triple positive cells and the mean protection induced at 1 month post-challenge were assessed using the Pearson correlation analysis.

## Results

### Characterization of vaccine-induced protective immunity

To assess whether the *ΔmmaA4*BCG deletion mutant formulated with or without the DDA/TDB adjuvant induced superior anti-tuberculosis protective immunity, the *ΔmmaA4* BCG preparations were tested in a mouse model of pulmonary TB. For the initial studies, mice were vaccinated once subcutaneously with either 10^6^ CFU of BCG Pasteur or the *ΔmmaA4* BCG mutant. Alternatively, mice were immunized three times with 10^6^ CFU of BCG Pasteur or the *ΔmmaA4* BCG strain suspended in the DDA/TDB adjuvant. At 2 months following the final vaccination, the mice were aerogenically challenged with a low dose of virulent *M. tuberculosis* Erdman. As seen in Experiment 1, all experimental immunization procedures induced significant levels of protective immunity (relative to controls) at 1 month post-challenge (Table 1). Moreover, the protective responses elicited by immunization with the BCG/adjuvant (−1.35 log_10_) or the *ΔmmaA4*BCG/adjuvant (−1.75) vaccine formulations were significantly enhanced compared to the live BCG control (−0.73).

**Table 1:** Evaluation of the anti-tuberculosis protective immunity induced by BCG strains formulated in DDA/TDB adjuvant at one month and four months post-challenge.

Experiment 1		
Group	1 Month	4 Months
Naive	5.44±0.04	
BCG	4.71±0.07 (−0.73)*	
BCGA4	4.37±0.19 (−1.07)*	
BCG/Adj	4.09±0.16 (−1.35)* #	
BCGA4/Adj	3.70±0.18 (−1.74)* # ∧	

Experiment 2		
Naive	4.95±0.11	4.67±0.13
BCG	3.95±0.10 (−1.00)*	3.77±0.19 (−0.90)*
BCG (3×)	4.05±0.12 (−0.90)*	ND
BCGA4	3.94±0.21 (−1.01)*	3.68±0.06 (−0.99)*
BCG/Adj	3.60±0.25 (−1.35)*	2.43±0.25 (−2.24)* # ∧
BCGA4/Adj	3.39±0.15 (−1.56)* # ∧	2.55±0.23 (−2.12)* # ∧

Experiment 3		
Naive	4.61±0.14	4.74±0.20
BCG	3.63±0.15 (−0.98)*	3.64±0.13 (−1.10)*
BCGA4	3.68±0.06 (−0.93)*	3.80±0.16 (−0.94)*
BCG/Adj	2.86±0.08 (−1.75)* # ∧	3.18±0.32 (−1.56)* ∧
BCGA4/Adj	2.45±0.19 (−2.16)* # ∧	3.07±0.11 (−1.67)* # ∧

BCGA4/Adj = *ΔmmaA4* BCG formulated in DDA/TDB adjuvant.

( ) The difference between naïve and experimental CFU.

ND – not done.

* Significantly decreased CFU values compared to naive controls, p<0.05.

# Significantly decreased CFU values relative to BCG, p<0.05.

∧ Significantly decreased CFU values relative to BCGA4, p<0.05.

In a second study, we evaluated the longer term effectiveness of these novel vaccine preparations. Again, at 1 month post-challenge, all of the vaccine formulations induced significant anti-tuberculosis protective immunity. Moreover, at the one month time point, immunization with the *ΔmmaA4*BCG/Adjuvant preparation (−1.56 log_10_) evoked significantly elevated protective responses compared to BCG alone (−1.00). Similar results were seen at 4 months post challenge. At this time point, all of the vaccine preparations were protective relative to naives (p<0.05). Furthermore, the *ΔmmaA4*BCG/adjuvant (−2.12) and the BCG/adjuvant (−2.24) formulations were strikingly more protective than BCG alone (−0.90). It should be noted that one group of mice was given BCG three times as a control in this study. Interestingly, three doses of BCG did not evoke increased anti-tuberculosis protective responses in mice at 1 month after the challenge when compared to a single BCG inoculation.

In a third study, vaccine-induced protective responses at one and four months following a tuberculous infection were again evaluated. At both time points, significantly decreased post-challenge CFU levels were seen in all vaccine groups relative to naïve controls (p<0.05). Additionally, immunization with both adjuvant-containing vaccine formulations (BCG/adjuvant, −1.75; *ΔmmaA4*BCG/adjuvant, −2.16) protected significantly better than BCG (−0.98) at 1 month post-challenge. Vaccination with the *ΔmmaA*4BCG/adjuvant mixture (−1.67) also yielded significantly enhanced protective responses relative to BCG controls (−1.10) at 4 months after an aerogenic challenge. Overall, in these three separate studies, immunization with the BCG and *ΔmmaA*4BCG-adjuvant formulations induced significantly increased anti-tuberculosis protective immunity compared to BCG controls in response to a tuberculous infection by the aerosol route.

**Figure 1:**
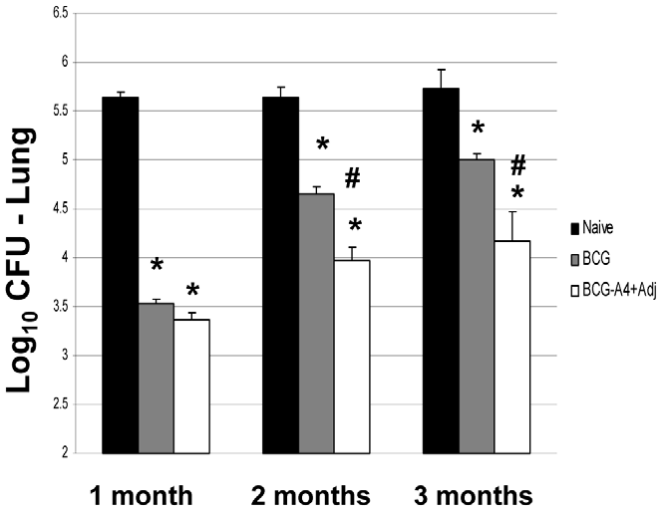
Protection in C57BL/6 mice against a *M. tuberculsosis* Erdman aerosol challenge after vaccination with either BCG or the *ΔmmaA4*BCG/adjuvant formulation. Mice were challenged with *M. tuberculosis* 8 weeks after a single s.c. immunization and then were sacrificed 1, 2 or 4 months after the challenge for enumeration of CFU's in the lung. Significant CFU reduction relative to * naïve mice (*p*<0.05) or ^#^ BCG-vaccinated controls (*p*<0.05).

In a fourth experiment, we tested whether significant anti-tuberculosis protective immunity could be generated in the lung with a single dose of the *ΔmmaA*4BCG mutant strain formulated in DDA/TDB adjuvant. As seen in Figure 1, a single dose of BCG or the *ΔmmaA*4BCG/adjuvant yielded substantial protection at one month post-challenge. However, at 2 months post-challenge, the *ΔmmaA*4BCG/adjuvant preparation induced significantly increased protection in the lung compared to BCG (−0.70 log_10_ difference). Similarly, at 4 months post-challenge, significantly better pulmonary protective immunity (−0.84 log_10_ difference) was detected in mice given a single dose of the *ΔmmaA*4BCG/adjuvant mixture relative to BCG.

### Analysis of vaccine-induced CD4 T cell immune frequencies by flow cytometry

**Figure 2:**
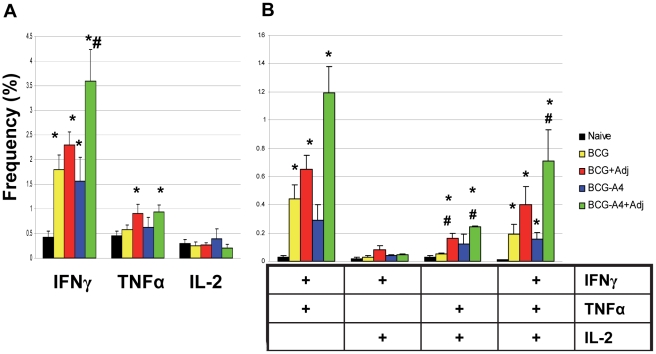
Muliparameter flow cytometry was used to determine the frequency (%) of (A) monofunctional CD4 T cells producing only IFNγ, TNFα, or IL-2 or (B) CD4 MFT cells producing both IFNγ and TNFα, IFNγ and IL-2, TNFα and IL-2 or all three cytokines from naïve or vaccinated mice. Splenocytes from three to five unchallenged mice per group were analyzed separately for these experiments. * Significant differences relative to naïve controls (*p*<0.05). ^#^ Significant differences compared to BCG-vaccinated mice (*p*<0.05).

Vaccine-induced T cell responses were evaluated using multi-parameter flow cytometry. At 2 months following the final immunization, spleen cells were removed from the vaccinated and naïve mice, stimulated overnight with BCG, stained for intracellular cytokines, and analyzed by flow cytometry. Initial analysis focused on CD4 T cell cytokine expression because CD4 T cells have been shown to be critical for controlling *M. tuberculosis* infections in the mouse model [24]. As seen in Figure 2, all vaccine preparations induced significantly increased frequencies of CD4 T cells expressing IFNγ, IFNγ/TNFα and IFNγ/TNFα/IL-2 relative to naïve controls. Interestingly, the frequencies of cells secreting either TNFα or TNFα/IL-2 were only elevated (compared to naïve controls) in spleen cultures recovered from animals vaccinated with the adjuvanted BCG vaccine preparations. To identify the cellular responses which may be responsible for the elevated levels of protection seen with the adjuvanted vaccines, the CD4 T cell frequencies induced by immunization with the adjuvanted preparations were compared to the cellular frequencies evoked by vaccination with BCG. Immunization with both the BCG/adjuvant and the *ΔmmaA*4BCG/adjuvant mixtures induced significantly higher numbers of double-positive CD4 T cells expressing TNFα/IL-2 than BCG (Figure 2B). Moreover, immunization with the highly active *ΔmmaA*4BCG/adjuvant preparation also induced elevated frequencies (relative to BCG) of double positive CD4 T cells expressing IFNγ/TNFα and triple-positive cells secreting IFNγ/TNFα/IL-2. To confirm these findings, additional experiments were done to evaluate CD4 T cell frequencies in BCG-vaccinated mice and mice immunized with the *ΔmmaA4*BCG/adjuvant preparation (Figure S1). Again, significantly higher frequencies of multifunctional CD4 T cells expressing TNFα/IL-2 and triple positive CD4 T cells were detected in splenocytes recovered from mice immunized with the *ΔmmaA4*BCG/adjuvant preparation compared to BCG-vaccinated mice.

**Figure 3:**
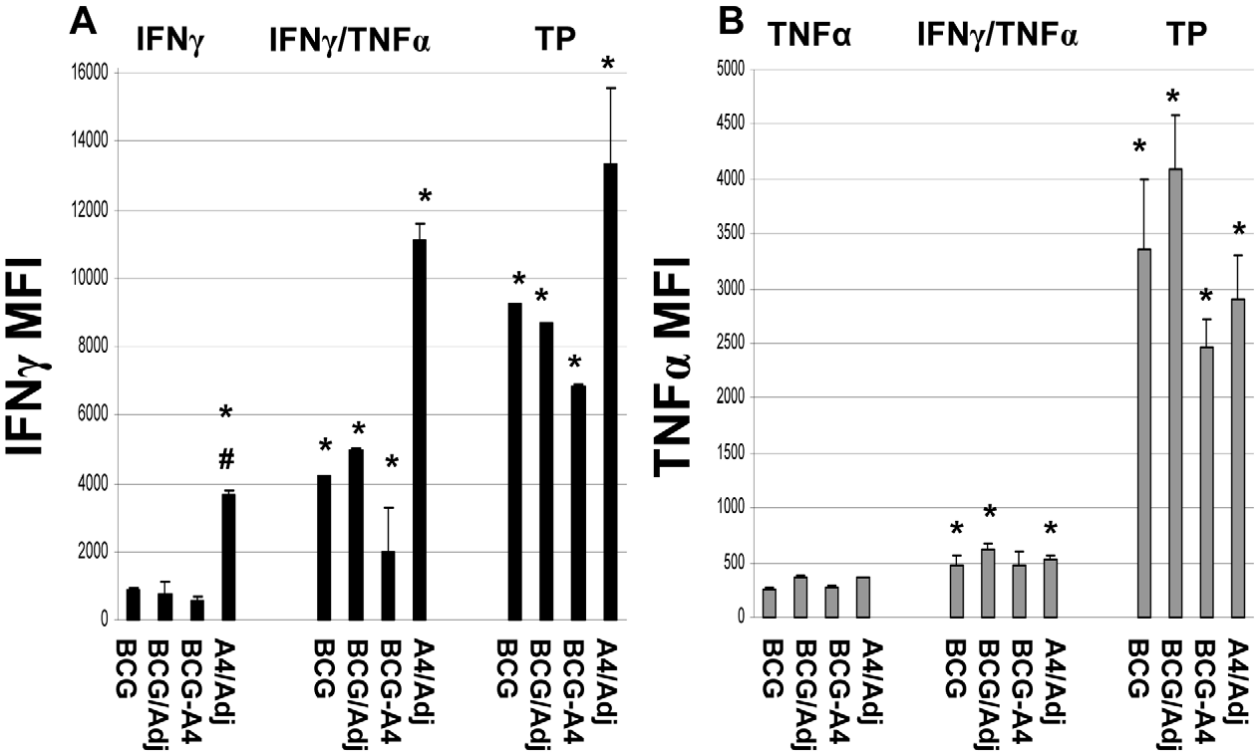
Median fluorescence intensity (MFI) of (A) IFNγ or (B) TNFα in monofunctional or multifunctional CD4 T cells. The data are presented as the mean of individual MFI values for 4–5 mice per vaccine group. * Significant differences relative to monofunctional cytokine expressing cells, *p*<0.05. ^#^ Significant differences relative to BCG in the same T cell subset, p<0.05.

Earlier studies have shown that CD4 T cells expressing multiple cytokines often produce higher amounts of cytokines per cell than monofunctional CD4 T cells [23], [25], [26]. To elucidate whether vaccine-induced MFT cells produced elevated concentrations of cytokines relative to monofunctional cells in these experiments, the median fluorescent intensities (MFI) for IFNγ and TNFα were determined for relevant cell populations evaluated in these studies (Figure 3A and B). However, for these experiments, the MFIs for IL-2 were not systematically assessed because generally low levels of IL-2 were detected in most studies. Consistent with previous data, elevated MFI values for IFNγ were detected in double-positive IFNγ/TNFα expressing CD4 T cells (3–7 fold increases) and triple positive CD4 T cells (3.5–12 fold increases) recovered from vaccinated mice compared to monofunctional IFNγ producing CD4 T cells from animals vaccinated with the same vaccines (Figure 3A). For these MFT cells, the highest MFI CD4 T cell values were again detected in mice vaccinated with the *ΔmmaA*4BCG/adjuvant mixture.

To further evaluate vaccine-induced immune responses, the total IFNγ response of the populations of cytokine-producing CD4 T cells was assessed using the integrated MFI metric (iMFI) which combines the magnitude and quality of T cell responses. Darrah et al have previously shown that iMFI values (frequency×MFI) for cells expressing IFNγ, TNFα, and IL-2 correlated with the protection induced by vaccines in a mouse model of *Leishmania* [25]. For our studies, the IFNγ iMFI values for IFNγ, IFNγ/TNFα, and IFNγ/TNFα/IL-2 producing cells induced by all of the vaccine formulations were elevated at least 10 fold relative to naïve controls (naïve iMFI data not shown). Furthermore, the IFNγ iMFI values were considerably elevated for the *ΔmmaA4*BCG/adjuvant immunization groups compared to BCG controls in each CD4 T cell subset evaluated. Increases relative to BCG iMFI values of 8.4-fold for IFNγ producing monfunctional cells, 7.2 fold for IFNγ/TNFα double positive cells and 5.3 fold for triple positive CD4 T cells were detected in splenocytes from *ΔmmaA4*BCG/adjuvant vaccinated mice (Figure S2A). Importantly, Pearson analysis showed that the correlation between vaccine-induced CD4 IFNγ iMFI values for triple positive cells and the mean protection induced at 1 month post-challenge shows a trend toward statistical significance (p = 0.07).

Analysis of the CD4 T cell MFI values for TNFα again demonstrated that multifunctional T cells express higher levels of cytokines than monofunctional cells (Figure 3B). The MFIs for TNFα were elevated about 2-fold for most experimental groups in IFNγ/TNFα expressing cells. Furthermore, striking 8–13 fold increased TNFα MFI values were detected in triple positive CD4 T cells relative to corresponding monofunctional cells. Also, as seen with IFNγ iMFI's, the iMFI values for TNFα were also significantly elevated in IFNγ/TNFα double positive CD4 T cells from *ΔmmaA4*BCG/adjuvant vaccinated mice relative to CD4 T cells from BCG-immunized mice (Figure S2B).

### Analysis of vaccine-induced CD8 T cell immune frequencies by multi-parameter flow cytometry

**Figure 4:**
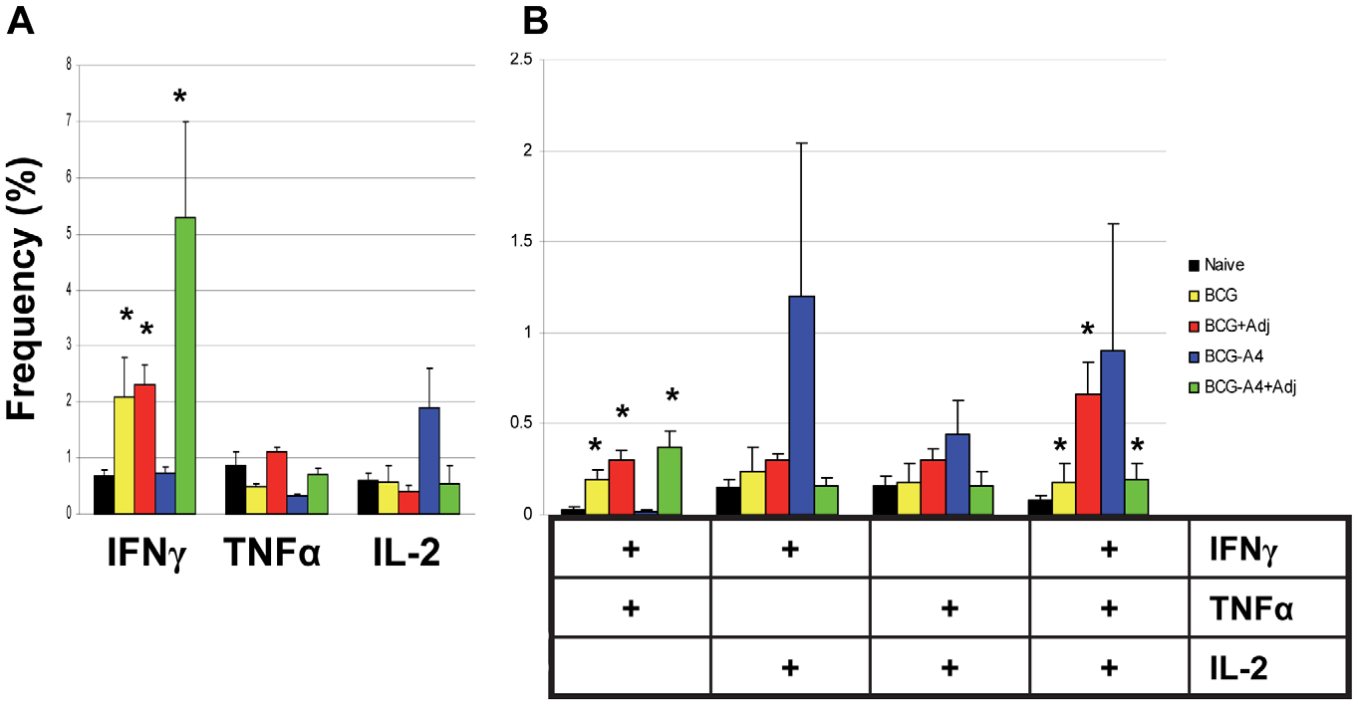
The frequency (%) of (A) monofunctional or (B) multifunctional CD8 T cells in naïve and vaccinated mice as determined by multiparameter flow cytometry analysis. * Statistical significance relative to naives, *p*<0.05.

Although CD4 T cells have been shown to be essential for controlling acute tuberculous infections in mice, CD8 T cells also play a role in limiting chronic murine TB disease [24], [27]. Using flow cytometry, we also analyzed vaccine-induced CD8 multifunctional T cell responses. As shown in Figure 4, immunization with the live vaccines with or without adjuvant induced significantly elevated frequencies of CD8 T cells expressing IFNγ (3–8 fold increases), IFNγ/TNFα (5–11 fold) and IFNγ/TNFα/IL-2 (3–9 fold) relative to naïve controls. However, no significant differences in vaccine-induced CD8 T cell frequencies were detected among the experimental groups.

**Figure 5:**
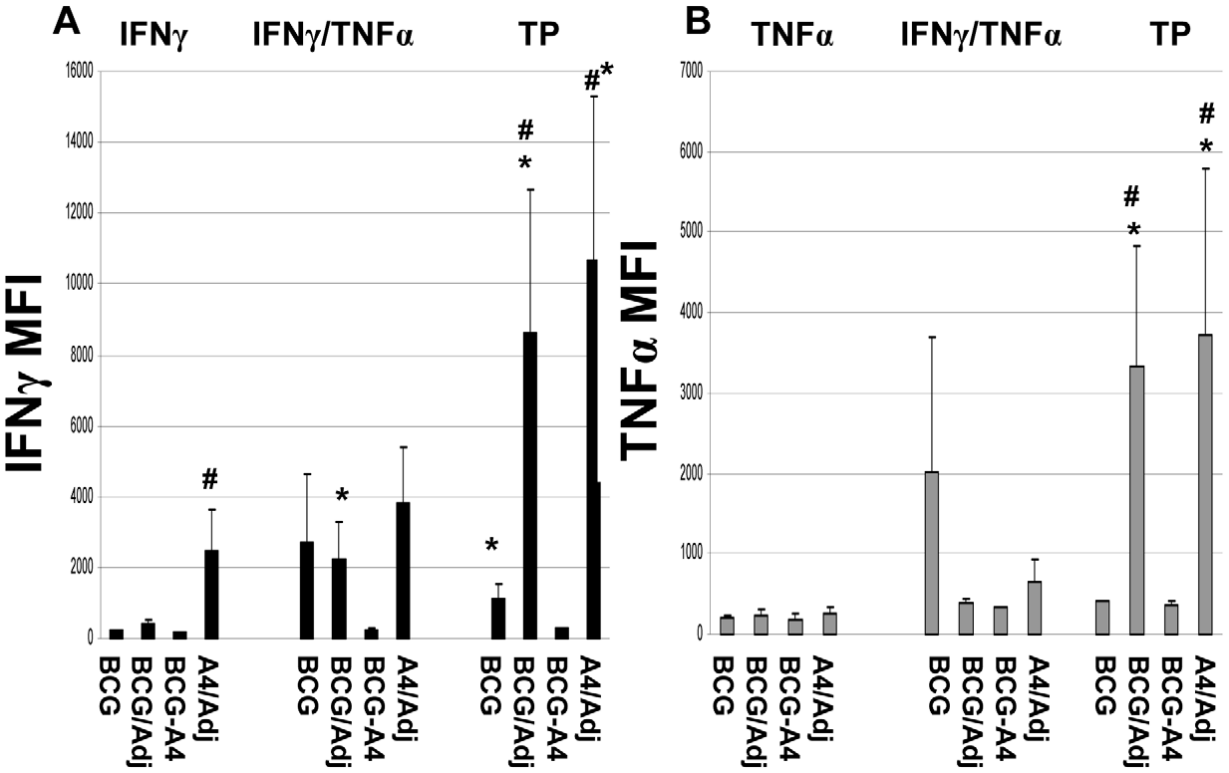
Median fluorescence intensity (MFI) of (A) IFNγ or (B) TNFα in monofunctional or multifunctional CD8 T cells. The data are presented as the mean of individual MFI values for 4–5 mice per vaccine group. *Statistical significance relative to monofunctional cytokine expressing cells, *p*<0.05. ^#^ Significant differences relative to BCG in the same T cell subset, p<0.05.

The CD8 T cell MFI for IFNγ in this study were generally consistent with the CD4 data; substantially higher MFI values were usually seen in vaccine-induced multifunctional CD8 cells than in cells expressing only IFNγ Figure 5A). For example, 5–20 fold increased IFNγ MFI values were detected in triple positive CD8 T cells taken from animals vaccinated with the BCG, BCG/adjuvant or *ΔmmaA*4BCG/adjuvant vaccines relative to corresponding monofunctional cell populations. Importantly, the IFNγ MFI values were increased by 7 and 9 fold, respectively, in the BCG/adjuvant and *ΔmmaA*4BCG/adjuvant preparations compared to BCG controls in triple positive CD8 T cells. Similar to the CD4 IFNγ iMFI results, elevated IFNγ iMFI values for the *ΔmmaA4*BCG/adjuvant vaccine group were detected in each CD8 T cell subset (Figure S3A). Compared to BCG controls, CD8 iMFI values were increased by 60-fold in monofunctional cells, 4.9 fold in IFNγ/TNFα producing T cells, and 33-fold in triple positive cells in splenocytes recovered from animals immunized with the *ΔmmaA4*BCG/adjuvant formulation.

Among the CD8 TNFα producing cells, the MFI values for the adjuvanted vaccines were only significantly elevated relative to nonadjuvanted BCG controls in triple positive cells (Figure 5B). The TNFα iMFI values detected in these triple positive cells were increased 30-fold for the BCG/adjuvant group and 15-fold for the *ΔmmaA4*BCG/adjuvant immunized animals compared to BCG controls (Figure S3B).

### Vaccine safety in immunocompromised mice

**Figure 6:**
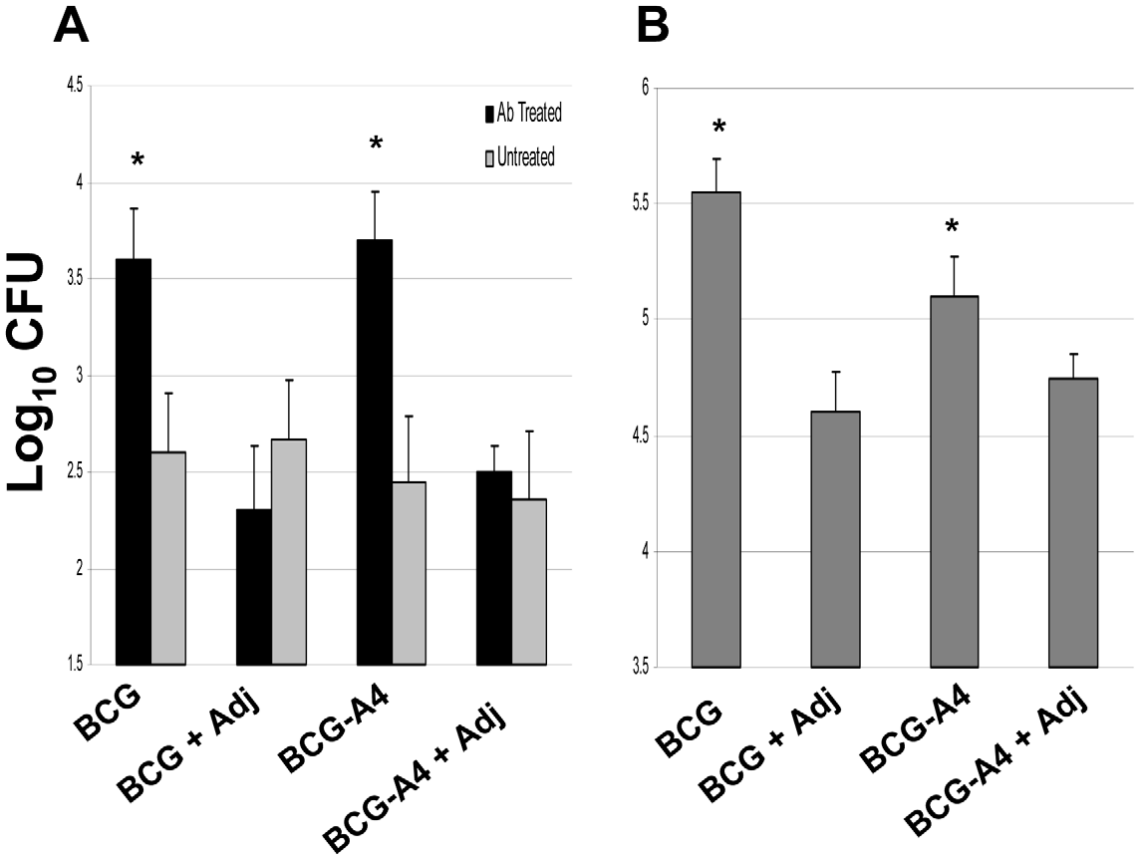
Reduced BCG splenic CFU levels one month after treatment of immunocompromised mice with the adjuvanted BCG preparations. A. Mice were given two i.p. injections of anti-IFNγ mAb (black bars) two days apart before receiving a single 10^6^ CFU dose of either BCG or *ΔmmaA4*BCG with or without adjuvant. Untreated mice served as infection controls (gray bars). The antibody treatment was repeated every 10 days until the mice were sacrificed one month post-infection to quantify the splenic BCG CFU. B. SCID mice were injected with a single 10^6^ CFU dose of either BCG or *ΔmmaA4*BCG with or without adjuvant. One month after the injection, splenic bacterial burdens were determined. *Significant differences relative to adjuvanted BCG controls, *p*<0.05.

The safety of immunizing HIV-infected children with live vaccines, and particularly BCG, has become an increasing public health concern [28], [29]. Since new TB vaccines will be used in some regions of the world with high HIV infection rates, the safety of adjuvanted BCG preparations was evaluated by assessing their capacity to proliferate in immunocompromised mice. In an initial study, the mice were treated with an anti-IFNγ antibody to reduce IFNγ concentrations and were then given one 10^6^ CFU dose subcutaneously of the control and adjuvant-containing mixtures. In the spleens of non-treated naïve mice at 1 month post-infection, nearly identical levels of BCG and the *ΔmmaA*4BCG mutant strain were detected in vaccine preparations formulated with and without the DDA/TDB adjuvant (Figure 6A). In contrast, significantly higher levels (>1 log_10_) of mycobacteria were detected in the spleens of the BCG and *ΔmmaA*4BCG immunized IFNγ depleted mice relative to the BCG/adjuvant and *ΔmmaA*4BCG/adjuvant infected immunodeficient mice at the 1 month time point (p<0.05).

To further assess the safety of the adjuvanted BCG preparations, SCID mice were injected with BCG or the *ΔmmaA4*BCG mutant as well as their adjuvanted vaccine counterparts. At 1 month post-vaccination, 5.55±0.06 log_10_ CFU of BCG vaccine and 5.13±0.08 log_10_ CFU of the *ΔmmaA4*BCG mutant were detected in the spleens of SCID mice (Figure 6B). In contrast, compared to the non-adjuvanted controls, significantly lower CFU (p<0.05) were recovered from the spleens of SCID mice vaccinated with the BCG/adjuvant preparation (4.58±0.07, −0.97 log_10_ reduction) or the *ΔmmaA4*BCG/adjuvant formulation (4.73±0.05, −0.40 log_10_ reduction).

## Discussion

In recent years, the BCG prime/TB antigen boost immunization strategy has been a prominent strategy for improving the effectiveness of vaccination against tuberculosis [11], [12]. While this approach is promising, it may be limited in developing countries by potential concerns about the cost of producing purified proteins, the safety of specific viral vectored vaccines (e.g. adenovirus), and the complexities of prime/boost immunization schedules. In this study, we have evaluated an alternative relatively simple and cost effective TB vaccination strategy. This approach combines the development of a mutant BCG strain designed to enhance Th1 immune responses with lipid formulation procedures [21]. We have shown in four separate experiments that a mutant *ΔmmaA*4BCG strain formulated in DDA/TDB adjuvant induced significantly elevated anti-tuberculosis protection relative to a BCG control at 1 and 4 months post-challenge. These results are consistent with studies in at least five different animal models which showed lipid encapsulation increased the effectiveness of BCG vaccine in protecting against tuberculosis [13]–[18].

The enhanced protection observed in our studies after immunization with the adjuvant containing vaccines was associated with the induction of increased levels of multifunctional T cells. In earlier studies, vaccine-induced amplification of MFT cells has been shown to correlate with protection against *L. major* and *M. tuberculosis* in mice as well as the control of SIV-viremia in non-human primates [23], [25], [26], [30]. Additionally, the presence of MFT cells is characteristic of immune responses seen in non-progressive HIV patients while HIV non-controllers generally elicit responses dominated by monofunctional cytokine cellular responses [31]. One characteristic of MFT cells that has been associated with their protective activity is their capacity to produce significant amounts of IFNγ and TNFα. Previous studies have demonstrated that vaccine-induced CD4 MFT cells express 3–10 fold more IFNγ and/or TNFα than corresponding monofunctional CD4 T cells producing only IFNγ or TNFα [23], [25], [26]. Our MFI data and iMFI calculations demonstrate that immunization with the *ΔmmaA*4BCG/adjuvant preparation and to a lesser extent the BCG/adjuvant formulation induces substantially elevated levels of these cytokines relative to BCG controls. Our statistical analysis, which showed that a trend in the correlation between iMFI values for triple positive cells and vaccine-induced protection (p = 0.07), suggests an important role for MFT cells in the protection mediated by the adjuvanted vaccines.

It is of considerable interest that both the *ΔmmaA*4BCG/adjuvant and BCG/adjuvant formulations induced significantly elevated levels (relative to BCG controls) of cells expressing both TNFα and IL-2. Recently, Lindenstrom et al demonstrated that vaccination with an Antigen 85-ESAT-6 fusion protein formulated in DDA/TDB adjuvant also elicited high levels of TNFα/IL-2 producing cells, a long-lived central memory cell population [32]. Orme has recently speculated that the Achilles heel of BCG is its inability to induce high numbers of central memory T cells [33]. Although BCG is very effective in inducing effector memory cells in lungs, it is relatively ineffective at evoking the more persistent, faster reacting central memory cells. The enhanced capacity of *ΔmmaA*4BCG/adjuvant and BCG/adjuvant vaccine formulations to induce elevated concentrations of the TNFα/IL-2 producing central memory T cells may contribute to the increased anti-tuberculosis protection seen after immunization with these preparations. Clearly, further studies are needed to evaluate the role of the TNFα/IL-2 expressing cells in the maintenance of long-term anti-tuberculosis cellular responses induced by BCG-containing vaccines.

The biological basis of the enhanced immunogenicity of the *ΔmmaA*4BCG/adjuvant vaccine is likely multi-factorial. First, the DDA/TDB adjuvant is known to be effective in generating Th1 and Th17 T cell responses [34], [35]. DDA is a synthetic amphiphile which forms cationic liposomes. Encapsulation of antigen within these liposomal structures can promote a depot effect leading to enhanced antigen persistence and increased monocyte influx into the injection site. The presence of the immunostimulatory TDB in the liposomes should also improve their drainage into the lymph nodes and enhance monocyte infiltration. Second, emulsifying BCG in the DDA/TDB adjuvant likely impacts the kinetics of BCG survival and the location of persisting BCG infections [15]. In oral vaccination studies, lipid encapsulation of BCG was shown to extend its persistence in vivo. Furthermore, formulating BCG in lipids may lead to more efficient delivery of live bacilli to sites of immune induction. Given that recent experiments have shown that clearance of *M. tuberculosis* by chemotherapy permits expansion of central memory T cells, changing the location and persistence of BCG by lipid encapsulation could significantly alter the relative proportion of vaccine-induced effector and central memory cells [36]. Our flow cytometry data, including the relative expansion of the central memory CD4 T cell subset expressing TNFα and IL-2 seen after immunization with the *ΔmmaA*4BCG/adjuvant vaccine, support this hypothesis. Surprisingly although the deletion of the *mmaA4* gene from *M. tuberculosis* has been shown in vitro to reverse repression of IL-12 production, we were unable to detect elevated splenocyte IL-12 message levels by RT-PCR after vaccination with the *Δmma4A*4BCG deletion mutant formulated with or without adjuvant. The reasons for the absence of *Δmma4A*4BCG vaccine induced IL-12 message expression are uncertain but may result because of differences between the *Δmma4A*4 *M. tuberculosis* and *Δmma4A*4BCG mutants or may reflect discrepancies between *in vitro* and *in vivo* experimentation. Alternatively, the increased IL-12 expression that has been previously observed in vitro [21] most likely occurs in microenvironments in vivo that may not be detectable by current methods.

Several recent published reports have shown that HIV-infected infants are highly vulnerable to disseminated BCG infections. Studies in South Africa and Argentina have indicated that the risk of vaccine-related disease after BCG immunization of HIV-infected children can be as high as one percent [28]. Consequently, the WHO has recommended that BCG should not be administered to HIV-infected children [29]. Surprisingly, in addition to being more immunogenic, we showed that the adjuvant containing BCG vaccines may also be safer. In immunocompromised mice that had been given identical doses of vaccine, approximately 5–10 fold lower concentrations of mycobacteria were detected in mice injected with the *ΔmmaA*4BCG/adjuvant and BCG/adjuvant formulations relative to non-adjuvanted controls. The mechanisms associated with the enhanced safety of the adjuvanted live vaccines in interferon-depleted animals and SCID mice are unknown but could result from either differential tissue sequestration or reduced proliferative rates of BCG suspended in the DDA/TDB mixture. Given that one-fourth of infants born in some African countries will likely be born to HIV-infected mothers, it is imperative that vaccine safety concerns related to BCG immunization be emphasized and fully evaluated as new TB vaccination strategies are being developed [37], [38].

In sum, we have shown vaccines prepared by mixing either BCG Pasteur or a *ΔmmaA*4 mutant BCG strain with DDA/TDB adjuvant yielded safer formulations that induced significantly more anti-tuberculosis protective immunity than BCG controls. With increasing pressure to develop inexpensive, safe, and more immunogenic TB vaccination strategies, further studies are urgently needed to confirm the increased safety and immunogenicity of these adjuvanted BCG formulations.

## Supporting Information

Figure S1 **To verify the multifunctional T cell frequency results that were observed in the initial experiments, a separate group of mice were vaccinated with BCG or BCG-A4/Adj and the frequencies (%) of CD4 (A) or CD8 (B) multifunctional T cells producing IFNγ and TNFα, IFNγ and IL-2, TNFα and IL-2 or all three cytokines (TP) were measured by flow cytometry.** Splenocytes from four unchallenged mice per group were analyzed separately. *Significant differences relative to naïve controls, *p*<0.05. ^#^Significant differences relative to BCG controls, *p*<0.05.
(TIF)

Figure S2 **The integrated MFI (iMFI) values for (A) IFNγ and (B) TNFα were calculated by multiplying the MFI values times the frequencies of IFNγ or TNFα single positive, IFNγ/TNFα double positive or triple positive (IFNγ/TNFα/IL-2) CD4 T cells.** iMFI values were derived from stimulated splenocytes from BCG, BCG/Adj. BCG-A4 or BCG-A4/Adj vaccinated mice. ^#^Significant differences relative to BCG controls, *p*<0.05.
(TIF)

Figure S3 **The iMFI values for (A) IFNγ and (B) TNFα are shown for IFNγ or TNFα single positive, IFNγ/TNFα double positive or triple positive (IFNγ/TNFα/IL-2) CD8 T cells using stimulated splenocytes from BCG, BCG/Adj. BCG-A4 or BCG-A4/Adj vaccinated mice.** ^#^Significant differences relative to BCG controls, *p*<0.05.
(TIF)
