# Supplementary figures and images for: Formulation of a mmaA4 Gene Deletion Mutant of Mycobacterium bovis BCG in Cationic Liposomes Significantly Enhances Protection against Tuberculosis
Source: PLoS One. 2012 Mar 19;7(3):e32959. doi: 10.1371/journal.pone.0032959 (PMC3307709; doi:10.1371/journal.pone.0032959)

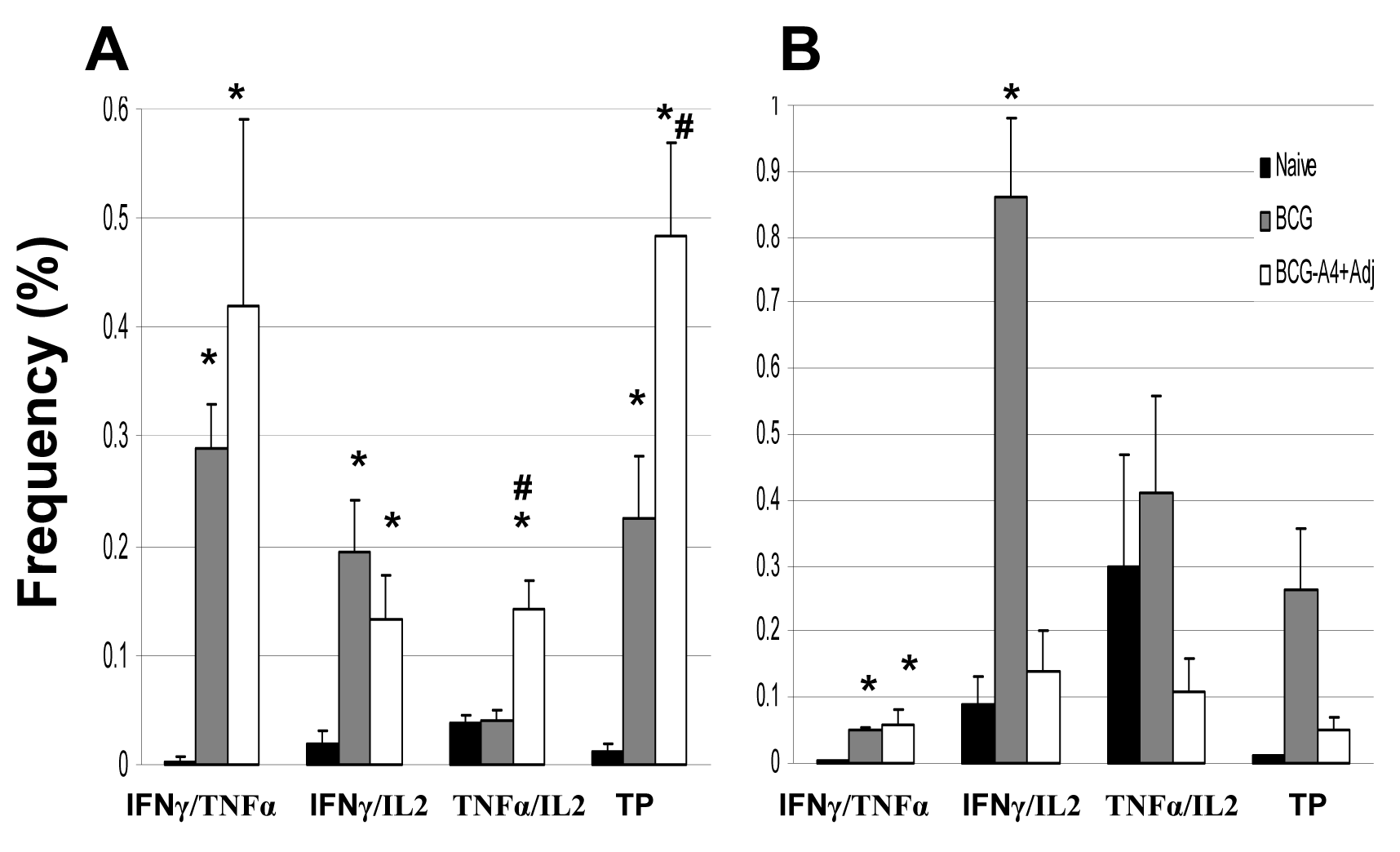

Supplement: Figure S1 — To verify the multifunctional T cell frequency results that were observed in the initial experiments, a separate group of mice were vaccinated with BCG or BCG-A4/Adj and the frequencies (%) of CD4 (A) or CD8 (B) multifunctional T cells producing IFNγ and TNFα, IFNγ and IL-2, TNFα and IL-2 or all three cytokines (TP) were measured by flow cytometry. Splenocytes from four unchallenged mice per group were analyzed separately. *Significant differences relative to naïve controls, p<0.05. #Significant differences relative to BCG controls, p<0.05. (TIF) [file pone.0032959.s001.tif]

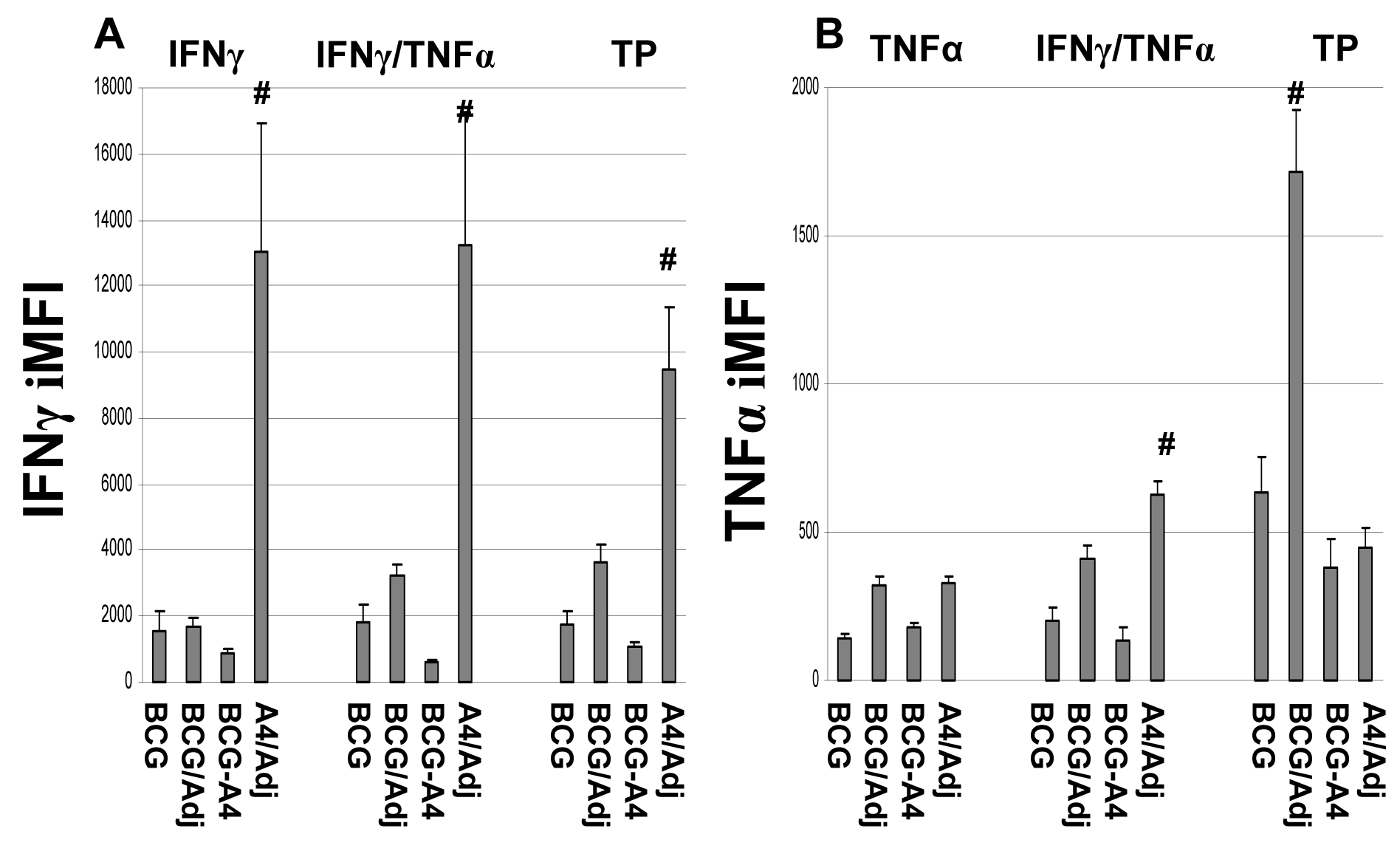

Supplement: Figure S2 — The integrated MFI (iMFI) values for (A) IFNγ and (B) TNFα were calculated by multiplying the MFI values times the frequencies of IFNγ or TNFα single positive, IFNγ/TNFα double positive or triple positive (IFNγ/TNFα/IL-2) CD4 T cells. iMFI values were derived from stimulated splenocytes from BCG, BCG/Adj. BCG-A4 or BCG-A4/Adj vaccinated mice. #Significant differences relative to BCG controls, p<0.05. (TIF) [file pone.0032959.s002.tif]

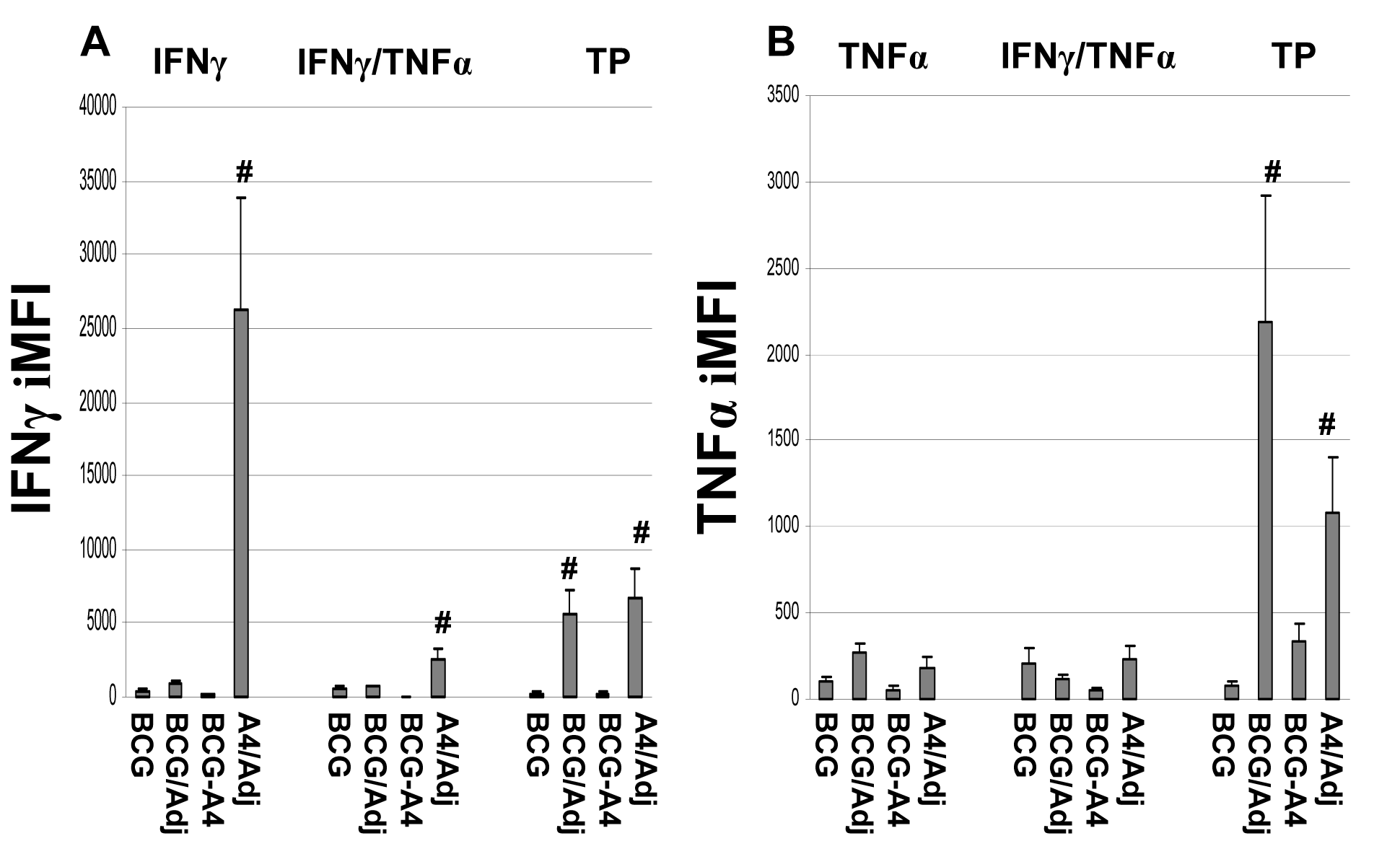

Supplement: Figure S3 — The iMFI values for (A) IFNγ and (B) TNFα are shown for IFNγ or TNFα single positive, IFNγ/TNFα double positive or triple positive (IFNγ/TNFα/IL-2) CD8 T cells using stimulated splenocytes from BCG, BCG/Adj. BCG-A4 or BCG-A4/Adj vaccinated mice. #Significant differences relative to BCG controls, p<0.05. (TIF) [file pone.0032959.s003.tif]
